# Characteristics and Performance Evaluation of QZSS Onboard Satellite Clocks

**DOI:** 10.3390/s19235147

**Published:** 2019-11-24

**Authors:** Wei Xie, Guanwen Huang, Bobin Cui, Pingli Li, Yu Cao, Haohao Wang, Zi Chen, Bo Shao

**Affiliations:** 1 College of Geology Engineering and Geomatics, Chang’an University, 126 Yanta Road, Xi’an 710054, China; chdxiewei@chd.edu.cn (W.X.); 2018126025@chd.edu.cn (Y.C.); mr.hao@chd.edu.cn (H.W.); 2019026017@chd.edu.cn (Z.C.); 2 German Research Centre for Geosciences (GFZ), Telegrafenberg, 14473 Potsdam, Germany; bobin.cui@gfz-potsdam.de; 3 Technische Universität Berlin, Str. des 17. Juni 135, 10623 Berlin, Germany; 4 The 20th Research Institute of China Electronic, Technology Group Corporation, 1 Baisha Road, Xi’an 710068, China

**Keywords:** QZSS satellite clocks, fitting precision, periodic terms, frequency stability

## Abstract

In the Global Navigation Satellite System (GNSS) community, the Quasi-Zenith Satellite System (QZSS) is an augmentation system for users in the Asia-Pacific region. However, the characteristics and performance of four QZSS satellite clocks in a long-term scale are unknown at present. However, it is crucial to the positioning, navigation and timing (PNT) services of users, especially in Asia-Pacific region. In this study, the characteristics and performance variation of four QZSS satellite clocks, which including the phase, frequency, frequency drift, fitting residuals, frequency accuracy, periodic terms, frequency stability and short-term clock prediction, are revealed in detail for the first time based on the precise satellite clock offset products of nearly 1000 days. The important contributions are as follows: (1) It is detected that the times of phase and frequency jump are 2.25 and 1.5 for every QZSS satellite clock in one year. The magnitude of the frequency drift is about 10^−18^. The periodic oscillation of frequency drift of J01 and J02 satellite clocks is found. The clock offset model precision of QZSS is 0.33 ns. (2) The two main periods of QZSS satellite clock are 24 and 12 hours, which is the influence of the satellite orbit; (3) The frequency stability of 100, 1000 and 10,000 s are 1.98 × 10^−13^, 6.59 × 10^−14^ and 5.39 × 10^−14^ for QZSS satellite clock, respectively. The visible “bump” is found at about 400 s for J02 and J03 satellite clocks. The short-term clock prediction accuracy of is 0.12 ns. This study provides a reference for the state monitoring and performance variation of the QZSS satellite clock.

## 1. Introduction

The performance of the atomic clock equipped on the navigation satellite plays a key role in the positioning, navigation and timing (PNT) services. In the domain of Global Navigation Satellite System (GNSS) measurement, the accurate position measurement is actually the precise time measurement. The time reference maintained on the navigation satellite is the onboard satellite atomic clock. The satellite clock error of 1 nanosecond (ns) is equivalent to about 0.3 meters distance measurement error [1]. The characteristics and performance of the satellite clock directly determine the accuracy of the PNT service and is beneficial to satellite clock offset prediction [2], satellite clock estimation [3] and integrity monitoring of the navigation system.

**Figure 1:**
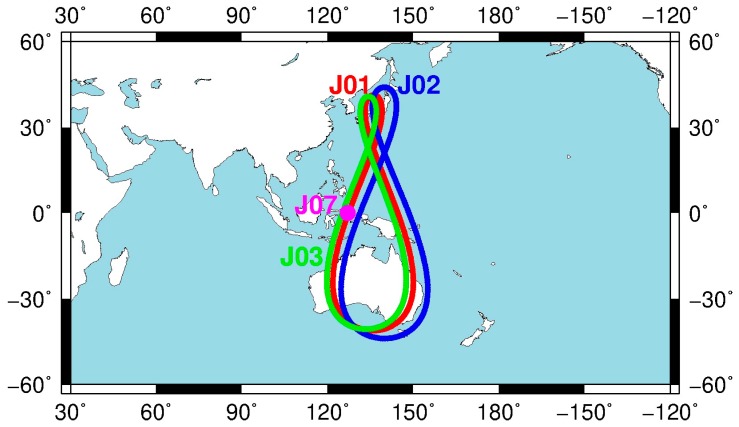
The ground track of four Quasi-Zenith Satellite System (QZSS) satellites.

The Quasi-Zenith Satellite System (QZSS) is an augmentation system of other satellite navigation systems [4]; its services were started officially on 1 November 2018 (http://qzss.go.jp/en/). At present, four navigation satellites have been launched. The information and ground track of four QZSS satellites are listed in Table 1 and Figure 1, respectively. As we can see from Table 1, the QZSS consists of three Inclined GeoSynchronous Orbit (IGSO) and one Geostationary Orbit (GEO) satellite. The first QZSS satellite, namely “Michibiki” was launched in 2010, and its service time is the longest among four QZSS satellites. The other three satellites were launched in 2017. The rubidium atomic clock is equipped on the QZSS satellite to maintain the time reference of satellite. It is noted that the rubidium atomic clock of QZSS is the same as the rubidium atomic clock equipped on the Global Positioning System (GPS) Block IIF satellites [5,6]. From Figure 1, the three IGSO satellites describe figure-of-eight loops, while one GEO satellite is fixed in about 127° E longitude. The orbit period of the QZSS satellites is a sidereal day, about 23 h and 56 min and 4 s [7]. The service range of QZSS is the Asia-Pacific region. Furthermore, the QZSS is transmitted on four frequencies, namely 1575.42 MHz (L1C, L1C/A and L1-SAIF), 1227.60 MHz (L2C), 1176.45 MHz (L5) and 1278.75 MHz (LEX), respectively [8]. The function of QZSS includes GPS complementary service, GNSS augmentation service and messaging service [7].

In the previous study about the QZSS satellite clocks. Guo et al. [6] estimated the frequency stability of the QZSS J01 satellite clock by adopting the Allan deviations, and the frequency stability is about 1 × 10^−13^ when the integration time is 1000 s. Hauschild et al. [9] analyzed the short-term stability of the J01 satellite clock by using the Allan deviations, and the frequency stability value when the integration time is 1, 10, 100, 1000 and 10,000 s are obtained, respectively. Delporte et al. [10] analyzed the onboard satellite clock of J01 satellite, and the Allan deviation of J01 satellite clock at 100 s is about 4 × 10^−13^. Steigenberger et al. [11] determined the orbit and clock of J01 satellite based on the Cooperative Network for Galileo In Orbit Validation Element (GIOVE) Observation (CONGO). Li et al. [12] presented precise orbit and clock determination of J02 satellite by using new satellite metadata, and after it was used, the frequency stability of 20,000 s was improved. Overall, previous studies mainly focused on the frequency stability analysis of J01 or J02 satellite clock. Nevertheless, the characteristics and performance of four QZSS satellite clocks in a long-term scale are unknown at present.

In this study, we focus on the characteristics and performance evaluation of four QZSS onboard satellite clocks. The satellite clock performance index, which including the phase, frequency, frequency drift, fitting residuals, frequency accuracy, periodic terms, frequency stability and short-term clock prediction were presented and analyzed in detail.

After this introduction, this paper is organized as follows: the data collection and the methods of performance evaluation of the QZSS satellite clock are presented in Section 2. Then, the characteristics and performance of the QZSS satellite clock are analyzed in Section 3 according to the result of the performance index of the satellite clock. Thereafter, the frequency stability time series of 100, 1000, 10,000 s and sub-daily frequency stability are analyzed. The short-term clock prediction is also implemented in Section 4. Finally, in Section 5, the conclusions are provided.

## 2. Data and Methods

In this section. At first, data collection is presented. Then, the method of data preprocessing is described. Thereafter, the satellite clock offset, frequency accuracy, frequency stability, periodic terms and short-term clock prediction model are described, respectively. Finally, the data process flow is given.

### 2.1. Data Collection

There are five Analysis Centers (ACs) contributing to the QZSS precise satellite clock offset products at present, which include Center for Orbit Determination in Europe (CODE), GeoForschungsZentrum (GFZ), Japan Aerospace Exploration Agency (JAXA), Technische University (TU) and Wuhan University (WHU) [13]. The precise satellite clock offset of the abovementioned ACs is generated by Orbit Determination and Time Synchronization (ODTS) algorithm [14,15,16,17,18], and they are coupled to the precise orbit, which can be used for characteristics and performance evaluation of satellite clock [19]. The ODTS algorithm can be found in [20]. In order to select the QZSS precise satellite clock offsets products for satellite clock analysis. The number of clock offset files of each satellite is counted from 1 January 2017 to 1 September 2019, and the statistics results are shown in Table 2. It can be seen that except for the J02 satellite clock, the file number of other satellite clock of GFZ Multi-GNSS Experimental (GBM) is the most, and the number of J02 satellite clock file is only 10 fewer than that of the products CODE. As a result, the precise satellite clock offset products provided by GBM, from 1 January 2017 to 1 September 2019, with an interval of 30 s were selected to evaluate and analyze the characteristics and performance of the QZSS onboard satellite clocks.

### 2.2. Data Preprocessing

The QZSS onboard satellite clocks are easily influenced by the sunlight, space environment [21] and magnetic [22] when running in the space. Thus, outliers are usually included in the precise satellite clock offset products, which should be detected and deleted before the evaluation. The Median Absolute Deviate (MAD) method is used to detect the outliers, while it is suitable for frequency data to detect the outlier. Hence, the clock offset should be transferred into the frequency before the MAD method is used [23]. Since the precise satellite clock offset products of GBM are estimated daily, data preprocessing is also conducted daily. The MAD method can be expressed as follows [23]:

(1) $$MAD\;=\;Median\left\{\left|y_{i}-b\right|/0.6745\right\}$$

where, $y_{i}$ is the frequency value, Median{} is the function to calculate the median value of data. $b\;=\;Median\left\{y_{i}\right\}$, when $\left|y_{i}\right|>\left(b+n\cdot MAD\right)$(n was selected as 3 in this paper) [24], then $y_{i}$ is regarded as an outlier.

### 2.3. Satellite Clock Offset Model

The frequency drift of rubidium atomic clocks is remarkable. Accordingly, the parameters of the satellite clock offset model are comprised of the phase, frequency and frequency drift. The quadratic polynomial model is selected to fit the precise clock offset products daily [24], and the quadratic polynomial model can be expressed as [25,26]:

(2) $$x_{i}=a_{0}+a_{1}\left(t_{i}-t_{0}\right)+a_{2}{\left(t_{i}-t_{0}\right)}^{2}+\varepsilon_{i}$$

where $x_{i}$ is the clock offset, $a_{0}$, $a_{1}$, $a_{2}$ are the phase, frequency and frequency drift, respectively, $t_{i}$ and $t_{0}$ represent the observation epoch and reference epoch, respectively. After the phase, frequency and frequency drift are obtained, the satellite clock offset model value can also be obtained. $\varepsilon_{i}$ is the difference between the satellite clock offset value in the clock product file and the satellite clock offset model value of each epoch, which is called the fitting residuals. After the $\varepsilon_{i}$ is obtained, the satellite clock offset model precision can be obtained according to the $\varepsilon_{i}$, and it can be expressed as follows:

(3) $$RMS\;=\;\sqrt{\frac{1}{N}\overset{N}{\underset{i\;=\;1}{\sum }}{\left(\varepsilon_{i}\right)}^{2}}$$

where,  RMS is the fitting precision, N is the number of clock offsets data.

### 2.4. Frequency Accuracy Model

Frequency accuracy is an index that is used to describe the consistency of the measured value or the calculated value with respect to the ideal value, and it is one of the most significant indexes to reflect the characteristics and performance of satellite clocks. The calculation of frequency accuracy is likely to be influenced by frequency stability and measurement noise. As a result, the sample time should be selected as long as possible when calculating the frequency accuracy. The clock offset data of one day is used to calculate a frequency accuracy value in this paper [27], the frequency accuracy model can be expressed as:

(4) $$K_{T}\;=\;\frac{\sum_{i\;=\;1}^{N}\left(x_{i}-\bar{x}\right)\left(t_{i}-\bar{t}\right)}{\sum_{i\;=\;1}^{N}{\left(t_{i}-\bar{t}\right)}^{2}}$$

where, $K_{T}$ is the frequency accuracy value. N represents the number of clock offset. $x_{i}$ is clock offset, $\bar{x}$ represents the average value of clock offset, $i.e.,\bar{x}\;=\;\frac{1}{N}\overset{N}{\underset{i\;=\;1}{\sum }}x_{i}$, $t_{i}$ is time series, $\;\bar{t}$ denotes the average time, i.e., $\bar{t}\;=\;\frac{1}{N}\overset{N}{\underset{i\;=\;1}{\sum }}t_{i}$.

### 2.5. Periodic Terms Model

The periodic variation of satellite clock offset exists in the satellite clocks of GPS and the second generation of BeiDou Navigation Satellite System (BDS-2) [19,28,29,30]. The reasons for this are the influence of solar illumination and the period variation of the satellite orbit. The period of satellite clock offset has an influence on frequency stability and satellite clock offset prediction [28,29]. In this paper, the periodic terms detection can be divided into three steps: First, the trend term in clock offset is removed by using quadratic polynomial, and the fitting residuals can be obtained. Then, the relativity effect caused by the oblateness of the earth in the fitting residuals is removed [31], and the relativity effect can be expressed as follows:

(5) $$\Delta t_{J_{2},rel}\;=\;-\frac{3}{2}J_{2}\frac{a_{E}^{2}}{c^{2}}\sqrt{\frac{GM}{a^{3}}}sin^{2}i\cdot sin2u$$

where, $\Delta t_{J_{2},rel}$ is the relativity effect, $J_{2}$ is the oblateness of the earth, $a_{E}$ represents the mean radius of the earth, c is the speed of light in a vacuum. GM is gravity constant times mass of the earth, a is the semi-major axis of the satellite orbit. i and u are the orbit inclination and the argument of latitude, respectively. Finally, the Fast Fourier Transform (FFT) algorithm is used for the fitting residuals without the relativity effect to detect the periodic terms [32].

### 2.6. Frequency Stability Model

Frequency stability is used to describe the random fluctuation of atomic clock output frequency caused by noise. Hadamard variance is insensitivity to linear frequency drift, making it particularly useful for the frequency analysis of rubidium atomic clocks [23]. Compared with Hadamard variance, Overlapping Hadamard Variance (OHV) can increase the equivalent degree of freedom, and then improves the confidence of its estimation. Therefore, the OHV method is selected to estimate the frequency stability of QZSS satellite clocks. Based on the clock offset, the OHV algorithm can be expressed as [23]:

(6) $$H\sigma_{y}\left(\tau \right)=\sqrt{\frac{1}{6\tau^{2}\left(N-3m\right)}\overset{N-3m}{\underset{i\;=\;1}{\sum }}{\left[x_{i+3m}-3x_{i+2m}+3x_{i+m}-x_{i}\right]}^{2}}$$

where, N is the number of clock offsets data, $\tau_{0}$ is the sample interval of clock offset data, and $\tau_{0}\;$= 30 s, m is the smooth factor, τ is smooth time and $\tau \;=\;m\tau_{0}$, m is selected as 4, 34 and 340, therefore, τ is 120, 1020 and 10,200 s, which are used to represents the frequency stability of 100, 1000 and 10,000 s, $x_{i}$ is clock offset.

### 2.7. Short-Term Clock Prediction

The short-term clock prediction can also reflect the stability of the satellite clock, and it can be used for real-time applications such as real-time precise point positioning (RT-PPP) [33,34]. Therefore, short-term clock prediction is also conducted. For each day, the precise satellite clock products are divided into six sessions and each session is four hours. The satellite clock offsets of the first two hours are adopted to fit by using the linear least square method, and the clock offset of the next two hours are predicted [35]. The prediction method can be expressed as follows:

(7) $$x_{i}=a+b\left(t_{i}-t_{0}\right)$$

where $x_{i}$ is the clock offset, both the a and b are the fitting coefficient. Furthermore, the Standard Deviation (STD) is used to assess the clock prediction accuracy. The concrete calculation method of prediction accuracy assessment is referred to as Huang et al. [30].

### 2.8. Data Process Flow

**Figure 2:**
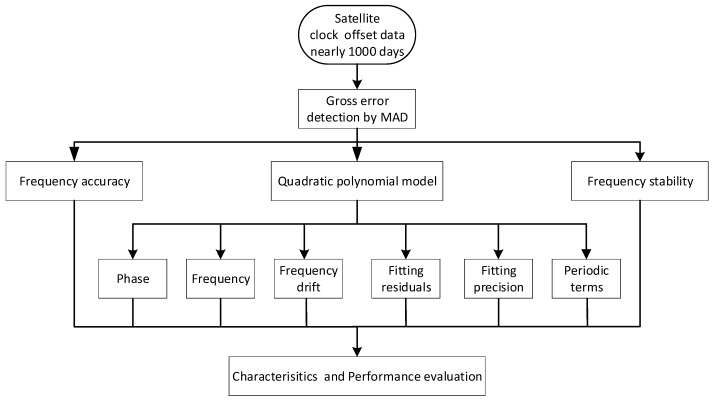
The clock offset data process flow.

Figure 2 depicts the data process flow of the characteristics and performance evaluation of the QZSS satellite clock. As we can see from Figure 2, the satellite clock offset products were obtained firstly. Then, the MAD method is used to detect the outliers existing in clock offset, which is significantly important to ensure the accuracy of all indexes of the QZSS satellite clocks. Thereafter, the clock offset data process was divided into three parts: First, the frequency accuracy is obtained by using the Equation (4) based on the preprocessed clock offset data. Second, the quadratic polynomial model is used to fit the clock offset daily; the phase, frequency, frequency drift, fitting residuals time series as well as fitting precision were acquired. After the relativity effect in fitting residuals is removed, the periodic terms are obtained by using the FFT algorithm. Third, the frequency stability is achieved by using Equation (6) based on preprocessed clock offset data. Therefore, all indexes of characteristics and performance have been calculated. Finally, we can evaluate the characteristics and performance of the QZSS onboard satellite clocks according to the variation of the above indexes. Furthermore, considering that the sample interval of clock offset has little impact on the calculation of the phase, frequency, frequency drift, fitting residuals and frequency accuracy, the sample interval of calculation of Equations (2), (3) and (4) is set as 300 s in order to improve the efficiency of the solution. While the sample interval of calculation of Equations (5), (6) and (7) are set as 30 s.

## 3. Analysis of Satellite Clock Characteristics

In this section, the quadratic polynomial model is used for the preprocessed clock offset data, the phase, frequency, frequency drift and fitting residuals time series and fitting precision are obtained. By using the Equation (4), the frequency accuracy time series are acquired. FFT algorithm is used for fitting residuals without the relativity effect, and the periodic terms are achieved. The characteristics of QZSS satellite clock variation with the time going on are analyzed.

### 3.1. Phase Time Series

**Figure 3:**
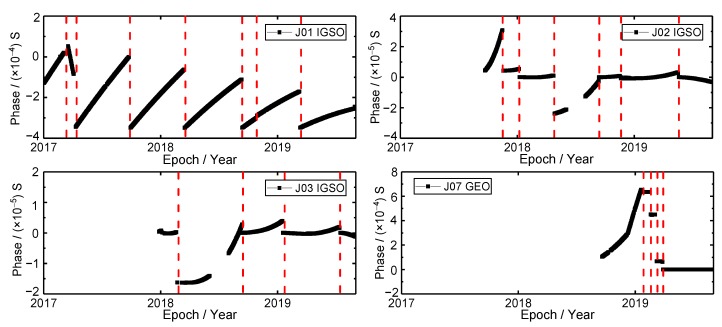
The phase time series of QZSS satellite clocks.

The phase time series are shown in Figure 3; the phase time series of the QZSS satellite clocks is smooth during the long period of operation. However, there are some phase jumps of the QZSS satellite clocks from 1 January 2017 to 1 September 2019. There are seven, six, four and four phase jumps that occurred in J01, J02, J03 and J07 satellite clocks, respectively (The red dash lines in Figure 3). The average time of phase jump is about 2.25 times for every satellite in one year. It is noted that there are frequent phase jumps of the J07 satellite clock in 2019. In general, there are two reasons for the phase jump. The first reason for phase jump is the switching of the satellite clocks. There are three atomic clocks equipped on each QZSS navigation satellite. If the main atomic clock is abnormal, the hot-backup clock should be turned on to replace the main clock [24]. The second reason is that the hardware is aging to a certain degree with the service time accumulated. In order to ensure the accuracy of the phase, it is necessary to calibrate the phase bias by modulating the phase. Thus, the phase jump occurs. The phase jump will result in the stability and continuity of satellite clocks to deteriorate. 

It is noted that there is a phenomenon that the discontinuity of phase time series occurred in J02 and J03 satellite clocks from June to August 2018. According to the website of QZSS (http://qzss.go.jp/en/), the orbit anomaly that occurred in the J07 satellite on 2 June 2018 accounts for this discontinuity phenomenon. The same anomaly occurred in J02 and J03 satellite as well, thus the signal transmission is suspended temporarily. The precise satellite clock offset products of the J07 satellite are provided by GBM since 18 September 2018, therefore, the signal interruption of the J07 satellite is not shown in Figure 3. The problem of orbit anomaly has been resolved later and three satellites were re-started on 31 July 2018. The discontinuity of phase time series will result in the time services discontinuity and poor accuracy of time synchronization [24].

### 3.2. Frequency Time Series

**Figure 4:**
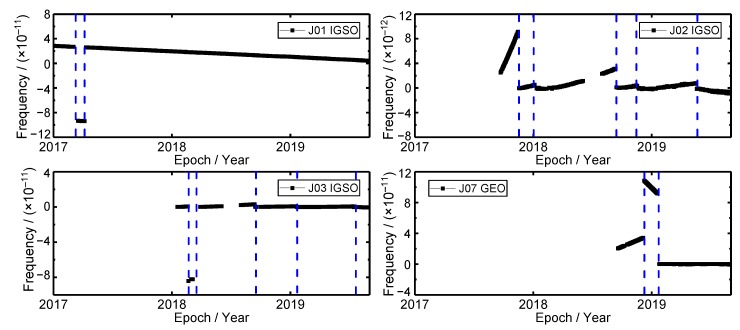
The frequency time series of QZSS satellite clocks.

The frequency time series of the QZSS onboard satellite clocks are shown in Figure 4. It can be seen that there are some frequency jumps occurring in each satellite clock. The J01, J02, J03 and J07 satellite clock experienced frequency jumps of two, five, five and two times, respectively (The blue dash lines in Figure 4). The times of the average frequency jump of QZSS satellite clocks is 1.5 times for every single satellite in one year. There are three reasons for the frequency jump. The first reason is that the frequency jump led by the phase jump. For instance, there are two frequency jumps occurred in J01 satellite clock in March 2017 and April 2017, respectively. The phase jumps also occurred at the same time. Therefore, the frequency jump results from the phase jump. The second one is the frequency modulation by the master control station of QZSS to guarantee the output frequency accuracy of satellite clocks. The last one is the switching of satellite clocks [24]. The switching of the satellite clock may also lead to frequency jump. The frequency jump has an influence on frequency stability and the quality of PNT services of satellite clocks. There are two methods to eliminate frequency jump. On the one hand, the times of phase and frequency jump caused by master station should be decreased so that the times of frequency jump can be decreased. On the other hand, the time synchronization between the main and hot-backup atomic clocks needs to be more accurate; therefore, after the satellite clock switch, the frequency jumps can be avoided. The output signal of the onboard satellite atomic clock is based on the frequency output source, and the frequency drift of the rubidium atomic clock cannot be adjusted by someone [24]; considering the different characteristics and performance of different satellite atomic clocks, the change of frequency slope can reflect the switch of the clock. It can be seen that the frequency slope of the J02 satellite clock has been changed in November 2017 and May 2019, respectively. The frequency slope of the J07 satellite clock has been changed in December 2018 and January 2019, respectively. Therefore, it can be concluded that these two satellites have undergone the clock switching. Overall, 4 QZSS satellites switched 4 times atomic clocks in 33 months. The average time of satellite clock switch is 0.36 times for every satellite in one year. The switching of the satellite clock has an influence on Precise Orbit Determination (POD) and the calculation of broadcast ephemeris parameters.

### 3.3. Frequency Drift Time Series

**Figure 5:**
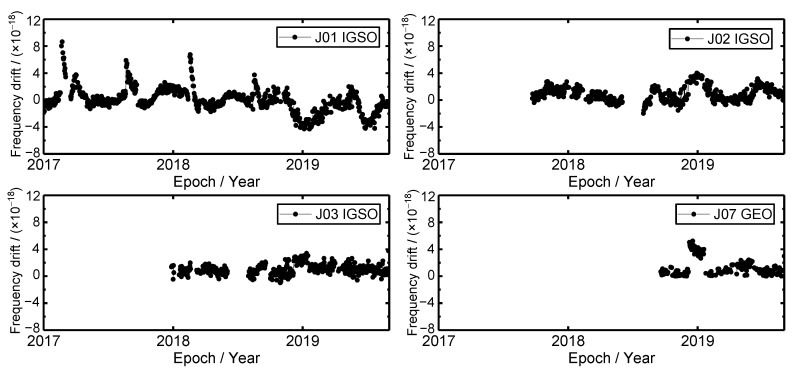
The frequency drift time series of QZSS satellite clocks.

Frequency drift is the rate at which atomic clocks change monotonously with running time. It can reflect the aging degree of atomic clocks. The frequency drift time series of the QZSS satellite clocks during 1 January 2017 to 1 September 2019 are shown in Figure 5. It can be seen that the frequency drift value of the QZSS satellite clocks is at a magnitude of 10^−18^. For the J01 satellite clock, there are four frequency drift jumps that happened in February 2017, August 2018, February 2018 and August 2018, respectively. The frequency drift value variations of these four frequency jumps are from 1.08×10^−18^, 1.30 × 10^−18^, 0.10 × 10^−18^ and 0.86 × 10^−18^ to 8.65 × 10^−18^, 5.89 × 10^−18^, 6.43 × 10^−18^ and 3.71 × 10^−18^, respectively. The frequency drift value sudden increases mean that they are abnormal at that time; after the frequency drift jump, the frequency drift value gradually decreases with the time goes on. It is noted that the phase jump occurs after the frequency drift jump. By combining Figure 3, one can know that after the frequency drift jump, a phase jump occurred, which indicates that the frequency drift becoming normal can be attributed to the phase modulation. Except for frequency drift jump, the variation range of frequency drift time series of J01 satellite clock is between −4 × 10^−18^ and 4 × 10^−18^. Furthermore, the periodic oscillation of the frequency drift of the J01 satellite clock is very apparent, and the period is about six months. For the J02 satellite clock, the frequency drift time series is very stable from September 2017 to June 2018, and the frequency drift value is in the range of −1 × 10^−18^ to 3 × 10^−18^. After repairing the malfunction of the J02 satellite, the frequency drift variation shows periodic characteristics, and the period is also six months, which is similar to the J01 satellite clock. Before and after the clock switch of the J02 satellite in May 2019, the fluctuation of frequency drift is from 2.08 × 10^−18^ to 2.68 × 10^−18^, which suggests that the onboard satellite clock switch of the J02 satellite has little effect on its frequency drift. Before the orbit abnormal of the J03 satellite, the frequency drift time series is stable; the frequency drift value is between −1 × 10^−18^ and 2 × 10^−18^. After the precise satellite clock offset product of J03 satellite is provided again, the frequency drift time series remains normal; the variation range of frequency drift is between −1 × 10^−18^ and 4 × 10^−18^. The frequency drift jump and periodic variation does not occur, which is not the same as the J01 and J02 satellite clocks. For the J07 satellite clock, the frequency drift experienced jumps in December 2018 and January 2019, respectively. The frequency drift value is from 0.54 × 10^−18^ to 5.03 × 10^−18^ and from 2.6 × 10^−18^ to 0.4 × 10^−18^ before and after the frequency drift jump, respectively. From figure 4, one can see that two frequency jumps occurred when the frequency drift jump, which implies that the frequency jump has an influence on frequency drift. Except for two frequency drift jumps, the variation trend of frequency drift of the J07 satellite clock is stable. Except for J03 and J07 satellite clocks, the periodic oscillation of J01 and J02 satellite clocks is remarkable. The reason for the periodic oscillation of frequency drift can be attributed to the periodic characteristics of satellite hardware device noises [24].

### 3.4. Fitting Residuals Time Series and Fitting Precision

**Figure 6:**
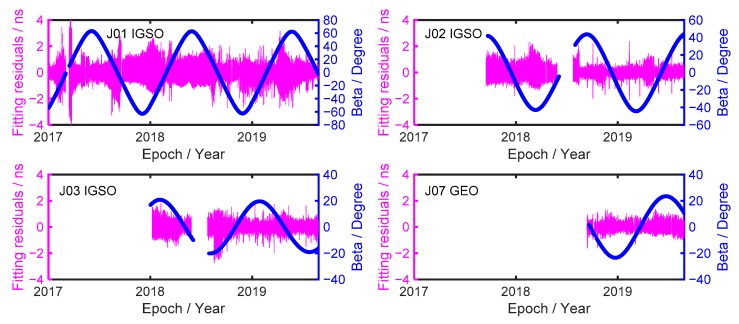
The fitting residuals time series of QZSS satellite clocks.

The fitting residuals, which are the difference between the clock offset value in the clock product file and the quadratic polynomial model value, can be used to reflect the noise level of the satellite clock. The fitting residuals time series of the QZSS satellite clocks are shown in Figure 6. Compared with the other three QZSS satellite clocks, the clock offset model noise of J01 satellite clock is the largest; its variation is from –4 to 4 ns, and the variation trend is irregular. As of January 2017, the service time of the J01 satellite is beyond six years, and its atomic clock is aging to a certain extent. The clock offset model noise was increased with the aging of atomic clock, which including random work frequency modulation (RWFM), flicker frequency modulation (FFM), white frequency modulation (WFM), flicker phase modulation (FPM), and white phase modulation (WPM), flicker walker frequency modulation (FWFM) and random run frequency modulation (RRFM). When the satellite clock offset modeling, the noise should be carefully considered. For the J02 satellite clock, before the malfunction, the fluctuation range of fitting residuals is between –2 and 2 ns. After the malfunction was repaired, the clock offset model noise becomes small, the variation range is from –1 to 1 ns. One can see that the clock offset model noise has been reduced, which means that the precision of the clock offset model is to be better. For the J03 satellite clock, before the malfunction of the J03 satellite, the fluctuation range of the fitting residuals gradually decreases, which implies that the precision of the satellite clock offset model gradually increases, and it is beneficial to satellite clock offset prediction. After the malfunction was repaired, the fitting residuals variation trend is stable. The fitting residuals of the J07 satellite clock are very stable, and there is no great fluctuation, its variation range is between –1 and 1 ns, which indicates that the precision of the satellite clock offset model is at a good state. Furthermore, except for J07 satellite clock, it is noted that the fitting residuals of J01, J02 and J03 satellite clocks suddenly increase, and after a period of time, about several days, the fitting residuals of these satellite clocks suddenly decrease. This was the case with J01 satellite clock in August 2017 and April 2019, J02 satellite clock in July 2018 and April 2019, and J03 in August 2018 and April 2019.

The yaw steering (YS) and orbit normal (ON) are adopted for the QZSS satellite. The β is an angle between the sun and the satellite orbit planes, also shown in Figure 6 (The blue solid line). For the |β| > 20°, the standard yaw-steering attitude of QZSS satellite is used. However, when |β| < 20°, the satellite attitude switches to orbit-normal orientation [36]. The switch of the QZSS satellite attitude has an influence on the accuracy of satellite orbit and clock solution. Prange et al. [37] has demonstrated that the RMS of fitting residuals of J01 satellite clock was degraded during ON model. For J01 the satellite clock, before 2018, when the fitting residuals increase, the |β| is less than 20°. Therefore, the large fitting residuals can be attributed to the switch of the satellite attitude. However, after January 2018, when the fitting residuals suddenly increase, the |β| is larger than 20° for J01, J02 and J03 satellite clocks, and the reason for large residuals cannot be attributed to the switch of the satellite attitude, which indicates that the orbit model of satellite attitude switch has been improved after January 2018. By combining Figure 3 and Figure 4, when the fitting residuals suddenly increase, the phase and frequency jump at the same time. Therefore, the reason for the fitting residuals suddenly increase is the frequent phase and frequency jumps.

The fitting precision, which is also called the satellite clock offset model precision, it can also reflect the noise level of the satellite clock offset model. According to Equation (3), the fitting precision is obtained. The fitting precision of the J01 satellite clock is 0.46 ns, which is consistent with the large fluctuation of its fitting residuals (see Figure 6). The fitting precisions of J02, J03, and J07 satellite clocks are 0.31, 031, and 0.24 ns, respectively. The fitting precision of the J01 satellite clock is larger than that of other QZSS satellite clock. The reason for this is that the service time of J01 satellite is longer than that of the other QZSS satellite. With the service times going on, the atomic clock is aging to a certain degree, and the clock offset model noise increases. Overall, the average fitting precision of the QZSS satellite clocks from 1 January 2017 to 1 September 2019 is 0.33 ns.

### 3.5. Frequency Accuracy Time Series

**Figure 7:**
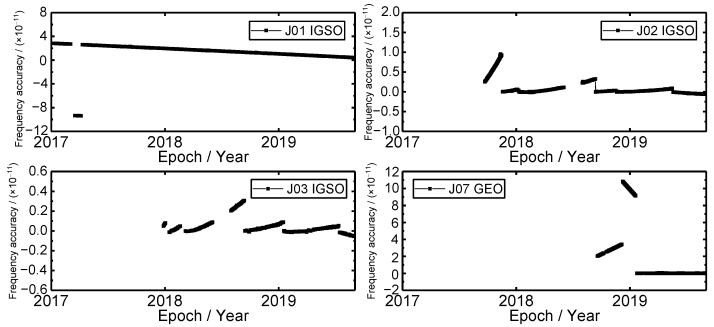
The frequency accuracy time series of QZSS satellite clocks.

The precise satellite clock offset product of each day is used to calculate a frequency accuracy value. Based on the Equation (4), the results were obtained and shown in Figure 7. It can be seen that the frequency accuracy of J01, J02, J03 and J07 satellite clocks show fluctuations to a certain degree. There are two, five, five, and two jumps that happened in J01, J02, J03 and J07 satellite clocks, respectively. By comparing the frequency accuracy and frequency time series, one can see that both the frequency and frequency accuracy have the same variation trend, and the times of frequency accuracy jump and frequency jump is also identical. Because the frequency accuracy can reflect the consistency between the measured frequency value and actually frequency value, and the output signal of the atomic clock is based on the frequency output source. The same variation trend of frequency and frequency accuracy means that the frequency accuracy is at a good state. Furthermore, for J02, J03 and J07 satellite clocks, the fluctuation of frequency accuracy is more frequent than that of the J01 satellite clock, especially at the early stage of satellite operation. The reason is that the satellite has started to operate, and the atomic clock has an aging process. The average frequency accuracy of J01, J02, J03 and J07 satellite clocks are 1.84 × 10^−11^, 9.54 × 10^−13^, 4.96 × 10^−13^ and 2.12 × 10^−11^, respectively. The frequency accuracy of J02 and J03 satellite clocks is two orders of magnitude higher than that of J01 and J07 satellite clocks.

### 3.6. Periodic Terms Results

**Figure 8:**
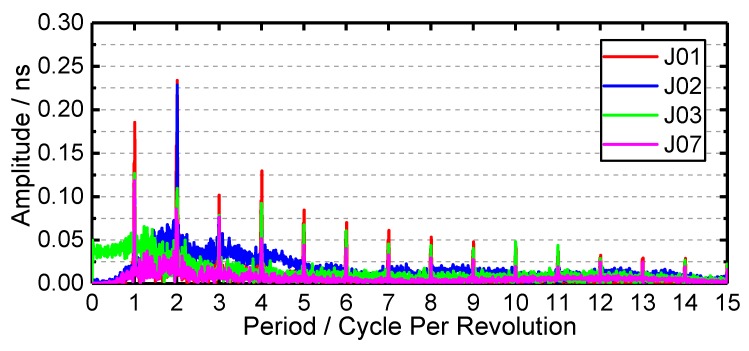
The amplitude spectrum of QZSS satellite clocks.

The amplitude spectrum of the QZSS satellite clock is shown in Figure 8. There are obvious periodic terms in all QZSS satellite clock offset data. For J01 and J02 satellite clocks, the amplitude of 2 cycles per revolution (cpr) is more evident than that of 1 cpr, and the main amplitude of these two satellite clocks is 0.234 and 0.228 ns, respectively. While for J03 and J07 satellite clocks, the amplitude of 24-h harmonic is more pronounced than that of 12-h harmonic, and the main amplitude is 0.127 and 0.118 ns, respectively. The 8-h harmonic (3 cpr) and 6-h harmonic (cpr) are also remarkable. Furthermore, except for J02 satellite clock, the n cpr (n is an integer) is also apparent; the amplitude decreases with the increase of n. Although the relativity effect in satellite clock offset is removed, the 2 cpr harmonic remains remarkable, which means that the reason for the clock offset period is not caused by the relativity effect. The main period of clock offset of two kinds of satellites is approximately 1 or 1/2 times of the orbital period of satellites. The orbit period is 23 h 59 min and 44 s. The period of satellite clock offset is slightly larger than that of orbit, and the ratio of satellite orbit period to clock offset period is 0.99725, which is consistent with GPS, BDS and Galileo [19,28,38]. As a result, some of the orbital error is absorbed by the clock offset when the satellite orbit determination and clock offset estimation simultaneously. The period of the QZSS satellite clocks offset is mainly influenced by the period of the satellite orbit. The characteristics of periodic signals is beneficial to the modeling of satellite clock offset and the satellite clock offset prediction that can be used for real-time applications such as real-time navigation.

## 4. Analysis of Frequency Stability

Because both the frequency stability and short-term clock prediction can reflect the clock stability. In this section, the frequency stability of 100, 1000 and 10,000 s of QZSS satellite clocks are obtained and analyzed. Furthermore, sub-daily frequency stability is also analyzed. Then, the short-term clock prediction of QZSS is conducted, and the clock accuracy is analyzed.

### 4.1. Frequency Stability

**Figure 9:**
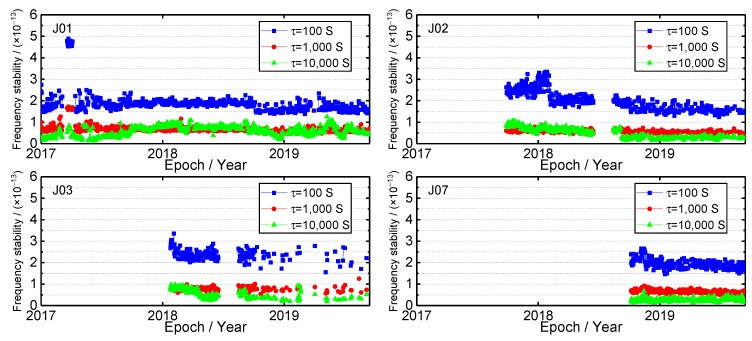
The frequency stability time series of QZSS satellite clocks.

The frequency stability time series of four QZSS satellite clocks are shown in Figure 9. For the J01 satellite, there are two frequency stability jumps that occurred in March 2017 and April 2017. The frequency stability value of 100, 1000 and 10,000 s are larger than that of other times at this period of time. From Figure 3 and Figure 4, one can see that there are two phase and frequency jumps at the same time. Therefore, it can be inferred that the frequency stability jump can be attributed to the phase and frequency jump. Furthermore, some fluctuations occurred in September 2018 and February 2019, respectively. From Figure 3, it can be inferred that the reason of fluctuations is the phase jump. Thus, the phase and frequency jump have an influence on frequency stability. The frequency stability time series of the J01 satellite clock is stable at the other times. As for J02 satellite clock, the frequency stability value is large before January 2018; especially for the frequency stability of 100 s, its variation range is between 2 × 10^−13^ and 3.5 × 10^−13^. After the phase and frequency jump, the variation trend of frequency stability time series becomes stable, which indicates that the phase and frequency jump has an effect on the frequency stability again. After the malfunction was repaired, the frequency stability value decreases, which means that the frequency stability of this satellite clock is to be better. For the J03 satellite clock, the frequency stability time series is stable before the malfunction. After repairing the malfunction, the fluctuation of frequency stability time series is larger, which can be attributed to the frequent phase and frequency jump at that time. For the J07 satellite clock, the frequency stability time series of 100, 1000 and 10,000 s continually decrease, and their value is smaller than that of other QZSS satellite clocks.

The average frequency stability when the integration time is 100, 1000 and 10,000 s of each satellite clock from 1 January 2017 to 1 September 2019 is shown in Table 3. When the integration time is 100, 1000 and 10,000 s, the J01, J02 and J07 satellite clock show the best performance among all QZSS satellite clock, respectively. For the QZSS, the average frequency stability of 100, 1000 and 10,000 s of four satellite clocks from 1 January 2017 to 1 September 2019 are 1.98 × 10^−13^, 6.59 × 10^−14^ and 5.39 × 10^−14^, respectively. The results are almost identical to Guo et al. [6]. For integration time of 100, 1000 and 10,000 s, the frequency stability of the QZSS satellite clock can fully meet its specifications [8]. For a different kind of satellite orbit, the frequency stability values of 100, 1000 and 10,000 s are 1.94 × 10^−13^, 6.51 × 10^−14^, 2.63 × 10^−14^ and 1.99 × 10^−13^, 6.60 × 10^−14^, 5.88 × 10^−14^ for QZSS GEO and IGSO satellite clocks, respectively. The frequency stability of GEO onboard satellite atomic clock is better than that of IGSO when the integration time is 100, 1000 and 10,000 s. Overall, the frequency stability QZSS satellite clock shows the superior performance. Considering that both the QZSS and the GPS Block IIF satellite clocks are the same atomic frequency standard [5,6], and the GPS satellite clocks have been demonstrated the good performance [25].

**Figure 10:**
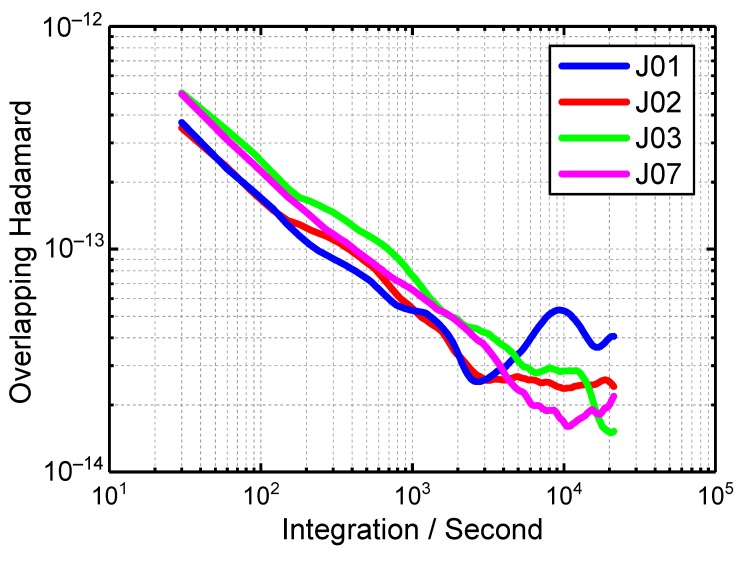
Sub-daily frequency stability of QZSS satellite clocks.

Except for the frequency stability time series, the sub-daily frequency stability analysis is also conducted. The sub-daily frequency stability of 30 January 2019 is shown in Figure 10. For the J01 satellite clock, the visible “bump” appears at about 1000 and 10,000 s, respectively. For the former “bump”, Steigenberger et al. [11] demonstrated that the 15 min periodic variations in the J01 clock, the reason for the “bump” at about 1000 s is the influence of the 15 min periodic variation. For the latter “bump”, the reason can also be attributed to the obvious periodic variation. From Figure 8, one can see that the period amplitude of the J01 satellite clock is the largest. However, the “bump” of J02 and J03 satellite clocks at 10,000 s are not more apparent than that of the J01 satellite clock. Both the J02 and J03 satellite clocks have a “bump” at about 400 s, which is the same as that of the J01 satellite clock of Steigenberger et al. [11].

### 4.2. Short-Term Clock Prediction

**Figure 11:**
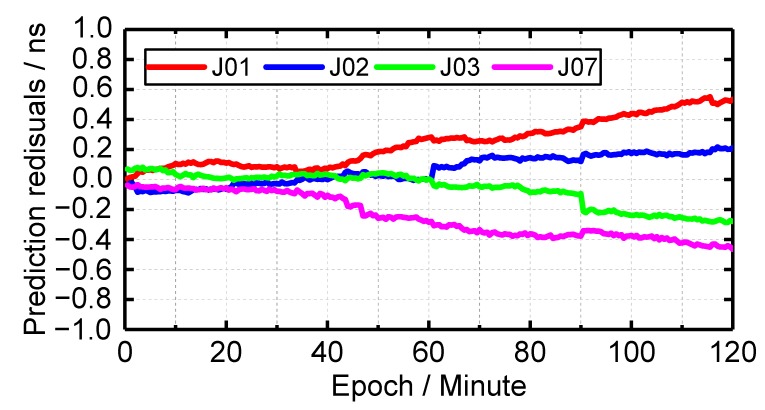
The prediction residuals of QZSS and BDS satellite clocks.

Because the clock accuracy assessment requires at least two satellite clocks of the same constellation, GFZ provide precise satellite clock offset products of two satellites from 21 September 2017. Therefore, the short-term clock prediction is conducted from 21 September 2017 to 1 September 2019. The short-term clock prediction residuals of one session are shown in Figure 11. One can see that when the clock prediction time is less than 50 min, the prediction residuals of all satellite clocks are less than 0.3 ns, and the prediction residuals are less than 0.5 ns when the clock prediction time is less than 100 min. When the clock prediction time is 120 min, the prediction residuals of J01, J02, J03 and J07 satellite clocks are 0.53, 0.21, 0.28 and 0.47 ns, respectively. The prediction residuals of J01 satellite clock is the largest among all QZSS, the reason for this is that the fitting residual is large (see Figure 6), which is bad for real-time application. The average short-term clock prediction accuracy of four QZSS satellite clock from 21 September 2017 to 1 September 2019 is 0.16, 0.10, 0.11 and 0.11 ns, respectively. which implies that the clock performance of J02, J03 and J07 satellite clock is better than that of J01. Overall, the short-term clock prediction accuracy of QZSS satellite clocks is 0.12 ns, which shows the superior performance of QZSS satellite clock again. 

## 5. Conclusions

The onboard satellite clock is the key equipment of the QZSS. The characteristics and performance of the onboard satellite atomic clock directly determine the performance of PNT services. In the GNSS community, the QZSS is an augmentation system in the Asia-Pacific region. In this contribution, the characteristics and performance of the QZSS satellite clock are presented and evaluated by using the QZSS precise satellite clock offset products provided by GBM from 1 January 2017 to 1 September 2019. The following conclusions can be drawn:

(1) The phase and frequency jumps are 2.25 and 1.5 times for every satellite in one year. The phase and frequency jump have an influence on frequency drift, fitting residuals and frequency stability. The magnitude of the frequency drift is about 10^−18^. The periodic oscillation of frequency drift of J01 and J02 satellite clocks is found, and the reason is that the influence of the satellite hardware device noise. The satellite clock offset model precision is 0.33 ns. Moreover, the fitting residuals variation is related to the *β*, phase and frequency jumps. The frequency accuracy is at a magnitude of 10^−11^ and 10^−13^.
(2) The period of 1 cpr and 2 cpr is more remarkable than other n cpr, and the 1 cpr is nearly the orbit period, which implies that the periodic terms in the clock offset is led by the orbit period. The periodic terms should be added to the ultra-rapid satellite clock prediction model so that the accuracy of the ultra-rapid clock prediction improves.
(3) The frequency stability of 100, 1000 and 10,000 s are 1.98 × 10^−13^, 6.59 × 10^−14^ and 5.39 × 10^−14^ for QZSS satellite clock. The visible “bump” is found about 400 s in J02 and J03 satellite clocks. The short-term clock prediction accuracy of QZSS satellite clock is 0.12 ns, respectively.

## Figures and Tables

**Table 1:** The information of the QZSS onboard satellite.

PRN	Launch Date	Orbit	Clock	Status
J01	11/09/2010	IGSO	Rb	Operational
J02	01/06/2017	IGSO	Rb	Operational
J03	09/10/2017	IGSO	Rb	Operational
J07	19/08/2017	GEO	Rb	Operational

**Table 2:** The file number of each QZSS satellite clock of different ACs. COD, GBM, JAX, TUM and WUM represents CODE, GFZ, JAXA, TU and WU, respectively.

	COD	GBM	JAX	WUM	TUM
J01	909	993	884	945	758
J02	657	647	76	238	454
J03	400	554	14	237	340
J07	0	315	0	223	17

**Table 3:** The frequency stability of QZSS satellite clocks.

PRN	τ=100 s	τ = 1000 s	τ=10,000 s
J01	1.86 × 10^−^^13^	6.78 × 10^−14^	5.41 × 10^−14^
J02	2.04 × 10^−^^13^	5.89 × 10^−14^	5.39 × 10^−14^
J03	2.36 × 10^−^^13^	7.64 × 10^−14^	5.35 × 10^−14^
J07	1.94 × 10^−^^13^	6.51 × 10^−14^	2.63 × 10^−14^
